# How do people feel while walking in the city? Using walking-triggered e-diaries to investigate the association of social interaction and environmental greenness during everyday life walking

**DOI:** 10.3389/fpsyg.2022.970336

**Published:** 2022-09-26

**Authors:** Lukas Bollenbach, Julian Schmitz, Christina Niermann, Martina Kanning

**Affiliations:** ^1^Department of Health and Social Sciences in Sport Science, University of Konstanz, Konstanz, Baden-Württemberg, Germany; ^2^Research Institute for Regional and Urban Development gGmbH, Dortmund, Germany; ^3^Medical School Hamburg, Institute of Interdisciplinary Exercise Science and Sports Medicine, Hamburg, Germany

**Keywords:** ambulatory assessment, mental health, active mobility, social interaction, greenness, momentary affective states, ecological momentary assessment, environmental factors

## Abstract

**Background:**

Light to moderate physical activity, which includes walking, is associated with positive effects on physical and mental health. However, concerning mental health, social and physical environmental factors are likely to play an important role in this association. This study investigates person-place interactions between environmental characteristics (greenness, social interaction) and momentary affective states during walking episodes. A within-subject design is implemented, in which affective states and environmental characteristics are assessed while participants are walking outside.

**Methods:**

On smartphones, coupled with a motion sensor (move3), e-diaries were triggered as soon as people walked 100 m outside. E-diaries assessed momentary affective states (valence, calmness, energetic arousal), and social interaction (walking alone; seeing other people while walking; interacting with other people; walking with a known person) between 6 am and 10 pm over nine days. The percentage of greenness was determined afterward from recorded GPS and GIS data. Demographics were collected in advance *via* an online questionnaire. Multilevel models were calculated with R for 46 individuals (age = 41.2, ± 13.2; 52% female).

**Results:**

Affective state dimension energetic arousal showed a significant association with social interaction and greenness, i.e., participants rated energetic arousal lower when walking alone, and if there was less greenness vs. when interacting shortly with someone while walking (*β* = 0.13, *p* = 0.02), and being in situations with more greenness (*β* = 0.08, *p* = 0.02). Furthermore, associations with social interaction and greenness were found for dimension calmness: walking together with someone was associated with higher calmness (*β* = 0.16, *p* = 0.02), and the higher the proportion of surrounding greenness during a walk, the higher calmness was rated, i.e., participants were calmer (*β* = 0.09, *p* = 0.01). Significant associations with valence were not present.

**Conclusion:**

The findings indicate that the affective states varied significantly due to different social and physical environmental factors. In the future, the importance of environmental factors should be further investigated, e.g., by assessing environmental factors right in situations contrary to a subsequent imputation. Within-subject designs, and in particular triggered assessments with the addition of GPS, can aid in developing interventions for health-promoting urban environments.

## Introduction

Walking, as a subcategory of physical activity (PA), representing low- to moderate PA, is associated with positive effects on individuals’ health and wellbeing (Ekkekakis et al., 2008a; Ettema and Smajic, 2015; Hanson and Jones, 2015; Wiese et al., 2018). Walking has been shown to enable individuals to achieve the WHO recommendations of a minimum of 150 min of moderate-intensity PA per week for health-enhancing effects, making it a valuable strategy for health promotion (Lee and Buchner, 2008; Wegener et al., 2017). While research regarding positive associations between walking and physical health is more consistent, this issue is more complex for mental health components like wellbeing. A closer look at study results regarding associations of PA and affective states shows that findings are not that clear and that moderating factors like the intensity of PA can play an important role: For example, it has been shown that PA levels exceeding a certain threshold can even have negative implications for individuals’ emotions and moods, and lead to the displeasure of PA (Ekkekakis et al., 2008b; Biddle, 2016; Brand and Ekkekakis, 2018). Furthermore, study results often stem from laboratory settings, neglect external social- and physical environmental factors (Liao et al., 2015), and/or do not consider within-subject changes in affect over time (variability of an individual’s affect over a period of time). However, that would provide more valid information about the association between walking, wellbeing, and possible moderators (Kanning et al., 2013). Therefore the goal of this study is to gain a better understanding of how affective states vary during everyday life walking episodes due to social and physical environment factors.

In line with social-ecological models, both social- and physical environments have to be taken into account to understand the association between PA and affective states, because individuals interact with- and are influenced by the surrounding environment (Guite et al., 2006; Helbich, 2018). A meta-analysis about exposure to nature showed positive effects on momentary affect: even brief contact and exposure to greenspace are associated with increased momentary wellbeing, as well as less negative and more positive affect (McMahan and Estes, 2015). In addition, it has been shown that urban greenspace that can be used (e.g., accessible parks, etc.) can have a protective effect on anxiety and mood disorder and that urban greenspace that can be observed (e.g., looking at it from home or work) can have a restorative effect (Nutsford et al., 2013). In the context of the various beneficial associations of environmental green with wellbeing and mental health, blue spaces must be mentioned as well. For example, they too have similar beneficial associations with wellbeing and mental health, and moreover have been associated with beneficial effects regarding recreation and stress-reduction that go beyond that of environmental green (Kistemann and Völker, 2014; Claßen and Bunz, 2018). Furthermore, environmental green and blue hasve been shown to be used for recreation, and to facilitate PA (e.g., walking) and social interaction (Sugiyama et al., 2008; Lachowycz and Jones, 2013; Grellier et al., 2017). An overview of evidence regarding associations of wellbeing and the physical environment shows that (urban) greenspace and also blue spaces can offer a place for and encourage recreational walking, PA, social cohesion, and facilitate social interaction among individuals, as they can bring people together (Newton, 2007; Grellier et al., 2017). Supporting these findings, Lachowycz and Jones (2013) developed a framework that highlights access to greenspace and its key moderator and mediator relationships regarding the interplay between individuals, the surrounding social- and physical environment, and wellbeing. More recent research also supports these associations that “(…) the presence of urban green spaces can encourage positive social interactions that cultivate social cohesion in ways that enhance health and well-being” (Jennings and Bamkole, 2019, p. 1). These research findings show the importance of the social context and that being socially well-integrated and having many social interactions can increase individuals’ wellbeing. Further underlining the previous findings, it was shown for both between- and within-subject associations that even brief and minor social interactions, e.g., greeting someone during a commute, promotes positive affect (Sandstrom and Dunn, 2014; Gunaydin et al., 2021). Furthermore, perceived social support can buffer against stress and negative affect (Smyth et al., 2014). In addition, social interactions, especially at social events during which individuals are physically active, showed a robust relation with high positive affect (Clark and Watson, 1988). Also, both weak and strong social ties and the social context in general (e.g., social cohesion and social interaction) are associated with individuals’ well-being, with improved social interactions inducing better wellbeing (Helliwell and Putnam, 2004; Helliwell, 2012; Schwanen and Wang, 2014). In conclusion, the results show that factors from the social- and physical environment play an important role in the relationship and context of everyday life walking routes and wellbeing and affect. To be more precise, the assumption is that at least part of the association depends on the context of walking, i.e., social- and physical environmental factors. In other words, it does matter where and with whom you walk, and this “where” and “with whom” influence the walking-wellbeing association.

In this regard, affective states are often used to describe how individuals feel in different social- and physical environmental contexts, as they have been shown to be an indicator of individuals’ wellbeing (Tost et al., 2019; Bourke et al., 2021; Monninger et al., 2022). In addition, affective states represent wellbeing in different situations, as they are more sensitive to the respective influences and external factors in the specific situation (Brose et al., 2013). Underlining this, walking in both urban- and natural environments has been shown to be associated with higher positive affect and energy (Kinnafick and Thøgersen-Ntoumani, 2014), and social interactions are associated with higher positive affect, more happiness, and less tiredness in daily life (Bernstein et al., 2018; Monninger et al., 2022).

However, dynamic assessments that integrate individuals and the surrounding environment are needed to gain a better understanding of these contexts (Helbich, 2018; Reichert et al., 2020). In this regard, real-time data assessment methods, such as ambulatory assessments (AA), have been proven to provide more accurate data of dynamic processes and environmental context-specific associations as well as affective states, compared to retrospective assessments methods that can produce biased data (Fahrenberg et al., 2007; Wilhelm and Schoebi, 2007; Ebner-Priemer and Trull, 2009; Dunton, 2017). Furthermore, to investigate health and behavior outcomes of complex momentary exposures in the (social- and physical-) environmental context, the addition of global position system (GPS) tracking has been shown to be a valuable add-on (Chaix, 2018; Reichert et al., 2021). In the context of this work, this means that by combining AA and GPS, it is possible to research momentary affect right in situations, in which individuals are walking outside, while also accounting for the specific environmental exposure in these situations. That the collection of data in an aforementioned way is feasible, has been shown by the authors of this paper (Kanning et al., 2022 (preprint)), and in addition, a better understanding of such time-varying relationships can provide further information about how to promote health-enhancing neighborhoods.

To our knowledge, no study has explicitly examined how social interaction and surrounding environmental greenness are associated with momentary affective states in walking situations in everyday life. One study did implement an illustrative smartphone design and examined physical (monotone–varied, dull–exciting), emotional (passive-active, sad–glad), and social (in company, social purpose of the trip) outcomes of walking, but only for a limited sample size of university students, focusing on safety and excitement issues, and without a triggered design (students were told to fill out a questionnaire either after walking for 5 min or retrospectively) (Ettema and Smajic, 2015). Contrary to the previously described data collection methods, incorporation of AA with GPS provides researchers with multiple additional benefits: It allows them to identify walking routes and implement corresponding event-based triggers, and to assess corresponding environmental features (e.g., greenness *via* subsequent imputation of Geographic Information System (GIS) data) in these situations, which enables comparison of different objective environmental features regarding their impact on affective states right in the corresponding situations. Extended knowledge about such event-specific associations between the environmental context and individuals’ wellbeing in everyday life is needed to aid in decision processes regarding the design of healthy places, where individuals reside. Therefore, this study implemented walking-triggered e-diaries to examine how social interaction and surrounding greenness are associated with individuals’ momentary affective states during walking episodes in everyday life. The following hypotheses, based on two predefined main effects, i.e., greenness, social interaction, and their association with affective states were formulated: in everyday life situations, in which individuals are walking outside, greenness and social interaction are positively associated with momentary affective states. In addition, we hypothesized an interaction effect between social interaction and greenness.

## Materials and methods

### Recruitment of the study participants

Subjects for this study were recruited in a two-step process. In a first step, 219 persons from several preselected urban residential areas answered a cross-sectional online questionnaire. In the second step, upon finishing the online questionnaire, participants were able to voluntarily participate in the AA by choosing one of the multiple available timeframes. Inclusion/exclusion criteria were as follows: age ≥ 18, no underlying physical- or mental health conditions (i.e., no restraints preventing them from being physically active, depression, etc.), an understanding of the German language, and living in a (sub-)urban residential area. A personal movement profile and an incentive of 50€ per participant were offered for participation through to completion. Participants had a mean age of 41.2 ± 13.2, and 52.17% were female, Table 1 depicts the characteristics of the study participants.

**Table 1:** General characteristics of the study participants and data included in the analysis.

*Descriptive statistics*	
Participants	*N* = 46
Sex	52% female
Age	*M* = 41 (± 13) years
Height	*M* = 175.2 (± 7.3) cm
Weight	*M* = 71.71 (± 13.9) kg
Education Level (higher school certificate)	81%
*Prompts*	
Average prompts/assessment period	8.5
Average steps prior to prompt	*M* = 193 (± 95)
Compliance	65.5%
*Variables*	
Valence (1–6)	*M* = 5.2 (± 0.9)
Calmness (1–6)	*M* = 4.8 (± 1.0)
Energetic Arousal (1–6)	*M* = 4.6 (± 1.1)
Social interaction (1–4)	*Md* = 3
Greenness (in viewshed)	33.7% (± 32.2)

M, mean; Md, median; Parameters valence, calmness, energetic arousal (metric scale, value of 1–6) and social interaction (ordinal scale, value of 1–4) were self-rated, and greenness was calculated as the percentage of greenness in the participants’ viewshed at the location of the triggered e-diary, ranging from 0 to 100%.

### Study design

This study implemented walking-triggered e-diaries to assess participants’ affective states in everyday life during walking episodes (Kanning et al., 2022 (preprint)). We used a new AA-trigger approach not only ascertaining the subjects’ affective states in everyday life but also accounting for subjective social interaction intensity and objective environmental greenness during walking episodes. This was accomplished using a study design with an interconnected technical interface between a smartphone (for electronic diaries, GPS- and transmission tower location tracking) and a hip-worn accelerometer. The two devices were coupled *via* Bluetooth, using the movisensXS-App (movisens GmbH, 2022)^1^. The accelerometer (Move 3, movisens GmbH^2^) has an internal memory card, a sampling frequency of 64 Hz, and can capture movement acceleration and body positions within a range of ±16 g (movisens GmbH^2^). Furthermore, the Move 3 has the advantage of being validated for documenting body positions and movement acceleration (Anastasopoulou et al., 2014; Giurgiu et al., 2020). The e-diaries were programmed to only be triggered upon several conditions: (1) Whenever movement acceleration exceeded a predetermined threshold (movement acceleration > 0.1 g for at least 1 min); (2) participants’ location was identified as non-stationary (i.e., a 100 m radius of a central position was left). If conditions were met, the cell phone vibrated, made an acoustic sound, and displayed a prompt to fill out e-diaries. Prompts remained active until the participant answered or actively rejected answering (if not rejected: Sound/vibration duration: 10 s; display duration: 50 s; number of alarms/reminder: 5; maximum delay time: 20 s). It took about 1 min to fill out the prompted questionnaires. Data was collected between 6 am and 10 pm.

### Procedure

After participants finished the online questionnaire and opted in for participation in the AA, an initial phone call to reassure their wish to participate in the study was done. Participants received oral and written information regarding the study procedures before written informed consent was obtained. Full ethical approval for this study as part of the AMbit project was obtained from the University of Konstanz (IRB18KN010–004, October 29, 2018). Next, participants were contacted again *via* telephone on the day their personal package with study items (smartphone-sensor combination) was sent to them. Moreover, participants were instructed on how to use the study smartphone and accelerometers. In addition, the phone call was used to provide the individuals with further information about the approximate arrival of their study items, general information, how to access the introduction/usage video, and contact/support options. The monitoring period usually started on Monday and was conducted for nine consecutive days, to collect data on both weekdays and weekends (the first and the last day were not included in the analysis, resulting in seven consecutive days ultimately being included in the analysis). Participants were required to carry the study items with them at all times while being awake, aside from non-compatible activities (e.g., sleeping, showering, swimming). After completion of the study, the participants sent the study items back and received the incentives, along with a personal movement profile.

### Data processing

After the study, we checked for and excluded incomplete data (i.e., technical problems and missings like ID unassignable: *n* = 8, no GPS-data: *n* = 5, accuracy >20 m and no street network point available: *n* = 11). Ultimately, the data of 46 participants remained eligible for inclusion in the analysis. In the next step, the raw acceleration data from the Move 3 were downloaded and implemented in the manufacturer’s data software ‘DataAnalyzer’ (v.1.13.5; 1.13.7; movisens GmbH^3^). Next, data were processed in 1-min intervals by the software, and a bandpass filter (0.25 to 11 Hz) automatically removed unwanted data components (i.e., gravitational components, artifacts, sensor shocks, etc.). Non-wear time (wear-time < 7 days, < 8 h per day) was identified *via* the aforementioned software and reassigned as missing values (NAs). In the next step, the e-diaries and GPS data from the smartphones had to be allocated to the concomitant accelerometer data. This was done by uploading the smartphone data to the movisensXS browser-APP, where it was processed, and downloaded, to merge the smartphone and accelerometer data with the manufacturer’s software ‘DataMerger’ (v.1.8.0; movisens GmbH^3^). After merging the different data into the final combined dataset, it was implemented in R (R Core Team, 2021) and RStudio (RStudio Team, 2021) for statistical data analysis.

## Measures

### Momentary affective states

To assess affective states, a shortened version of the Multidimensional Mood Questionnaire was used (Steyer et al., 1997). The scale was developed and validated for use in AA studies and homogeneity of the scale items was evaluated by Wilhelm and Schoebi (2007) for both within- and between-subject levels and satisfactory internal consistency attested. It measures affective states with three dimensions (valence, calmness, energetic arousal). Further, the scale consists of 2 items per dimension that are ordered as semantic differentials and measures the intensity of the affective states (i.e., for valence: unwell-well, discontent-content; for calmness: relaxed-tense, calm-agitated; and for energetic arousal: tired-awake, without energy-full of energy). Participants answered the triggered prompts (“At this moment, I feel…”) on a 6-point-Likert scale with a left- (e.g., 1 - unwell) and right extreme (e.g., 6 - well), and the score for each dimension was derived by averaging the corresponding item scores. Using the aggregated data across the participants, Cronbach’s alpha was computed for each dimension, resulting in a value of 0.85 for valence, 0.86 for calmness, and 0.81 for energetic arousal in this study.

### Social interaction

**Figure 1:**
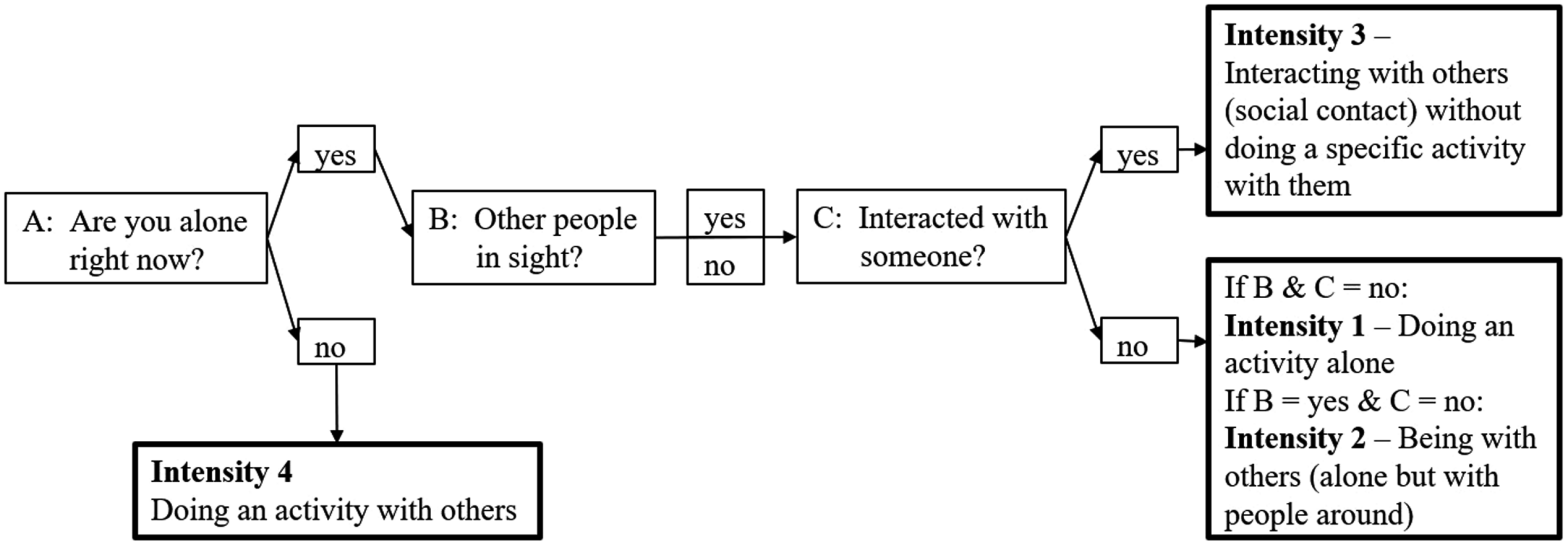
Overview of the 4 (in magnitude ascending) social interaction intensities (intensity 1–4) that are based on the taxonomy of social activities from Levasseur et al. (2010). Boxes with regular contour strength depict the prompted questions (A, B, C) and concomitant answer options, upon which the social interaction intensity was determined (bold boxes) (own depiction).

To collect data on social interaction, we developed an instrument to assess social interactions in daily life: It is based on the taxonomy of social activities from Levasseur et al. (2010) who propose 6 proximal to distal intensities of social activities, based on the concomitant goal: i.e., (1) doing an activity in preparation for connecting with others, (2) being with others, (3) interacting with others without doing a specific activity with them, (4) doing an activity with others (5) helping others, and (6) contributing to society (Levasseur et al., 2010). Since this study is researching affective states of individuals in situations of walking episodes, we decided to modify the taxonomy to better fit the circumstance of walking by describing the magnitude of interactions of the participants in 4 ascending intensities: (1) doing an activity alone (walking); (2) being with others (alone but with people around, i.e., someone is at least in sight); (3) interacting with others (social contact) without doing a specific activity with them (e.g., greeting someone); (4) doing an activity with others (we assume that an individual interacts with another when walking together). Figure 1 depicts an overview of the 4 intensities and the conditions for each.

### Greenness

**Figure 2:**
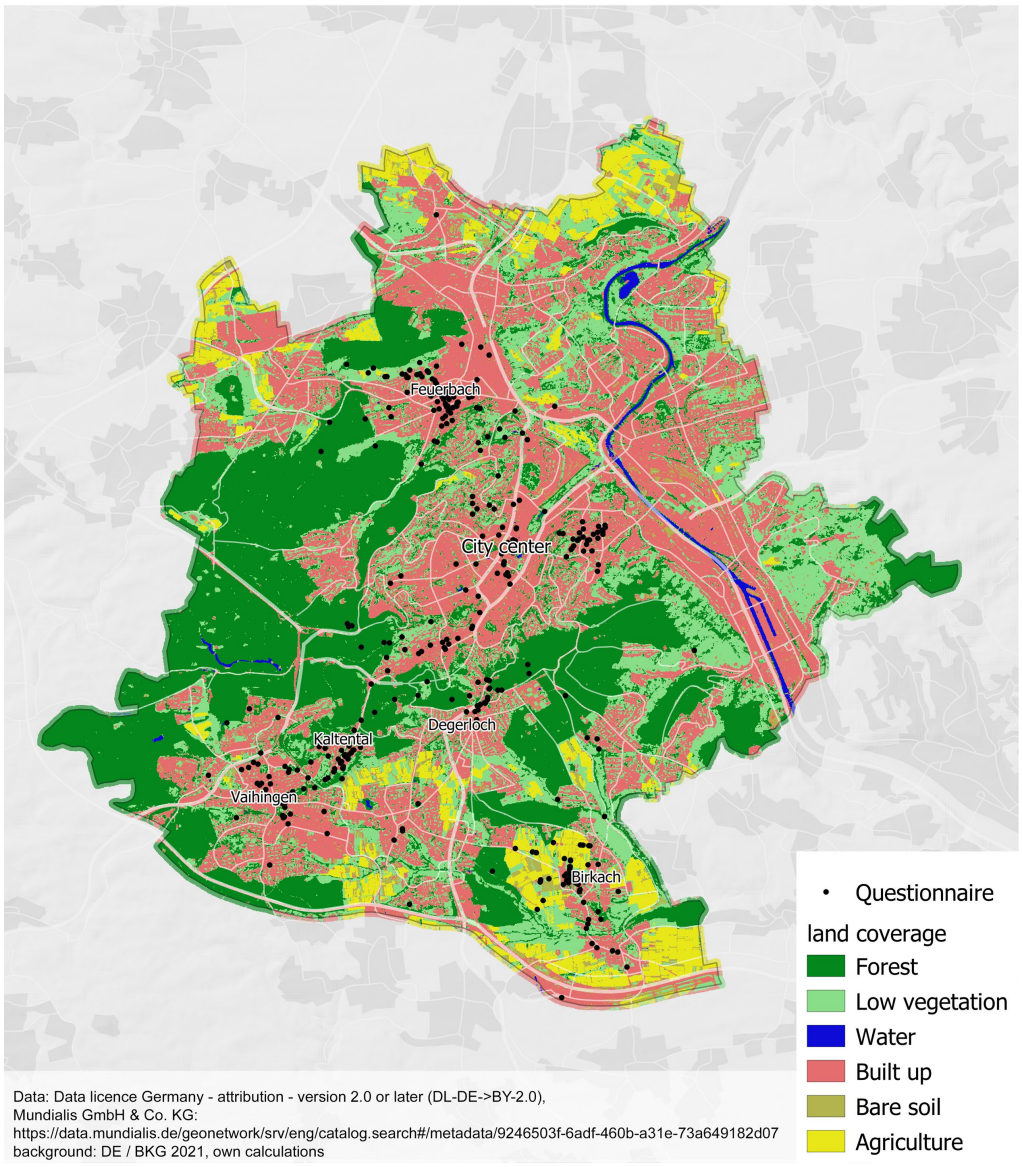
Study location, questionnaire locations, and the types of land coverage.

The greenness was determined *via* GIS as a percentage of the green and blue areas within the potential viewshed of the participants (= the area visible by a participant from a corresponding location) at the location of a triggered e-diary (= observation). In the first step, the trigger points were filtered and corrected in terms of location: Only locations within the city of Stuttgart with an accuracy of fewer than 20 m were included in the analysis. High accuracy of the location data is required for the calculation of the viewshed. If the location is too imprecise, buildings, for example, can significantly influence the viewshed. These observations were relocated to the nearest point of the street network of OpenStreetMap (OSM^4^) (maximum 30 m; OpenStreetMap contributors, 2015). The next step was to create a surface model to determine the viewshed. Chaix (2018) recommends focusing on a small scale (50 m or 100 m), which represents a viewshed. We pushed it forward and calculated a potential viewshed (Chaix, 2018; see also Tost et al., 2019). The viewshed of each location of the questionnaire (GPS) is bounded by buildings and the topography. The buildings from OSM were given a height of 8 m and were converted to a raster image. The European Digital Elevation Model was bilinearly resampled to 50 cm. The values of both raster images were added. Based on the position-corrected location and the surface model, a potential viewshed was calculated using the software ArcMap^5^ 10.6.1 by ESRI^6^ (ESRI, 2010). The maximum distance of sight is 100 m (Labib et al., 2020; Boakye et al., 2021). Smaller gaps within the viewshed, which were caused by the edges in the elevation model, were filled. The green areas (grassland, forest) including water bodies were extracted from a land cover classification based on Sentinel-2 data^7^ for the year 2020 with a 10 m × 10 m resolution (data license by-2-0, own calculation; mundialis GmbH & Co. KG, 2020). The classification was developed within the incora project (see BMVI, 2018).^8^ This classification that we used was the closest classification to the time of data collection, as participants were recruited from July 2020 until December 2020. It consists of the following landcover classes: forest, low vegetation, water, built-up, bare soil, agriculture (mundialis, 2021^7^). Each pixel is completely assigned to one landcover class and we used forest, low vegetation, and water as green and blue spaces. The sum of all 391 viewsheds included in the analysis is 4.373 km^2^. Of these, 2.371 km^2^ are made up of built-up, bare soil, and agriculture combined, 0.908 km^2^ are covered by forest, 1.091 km^2^ by low vegetation, and 0.003 km^2^ (or 0.17%) by water. The proportion of greenness results from the proportion of the viewshed that is covered with greenspace (mean: 33.79%, min/max: 0–100%; SD: 32.17). Labib et al. used a similar approach by using a digital surface model, a digital terrain model, and a land cover map to estimate greenness visibility (Labib et al., 2020). Figure 2 depicts the study location, the locations of the triggered questionnaires, and the types of land coverage.

### Covariates

The covariates for this study are derived from a self-report online questionnaire (comp. Recruitment and selection of study participants), which the participants filled out prior to the start of this study. Covariates consisted of participants’ demographics, i.e., age, sex, and educational level. The covariates age and sex were included in the models and also used to check for cross-level interaction effects (educational level was not included as over 80% of the study sample had at least a higher school certificate).

## Data analyses

To analyze the between- and within-subject relationships between affective states, social interaction intensities, greenness, along with the cross-level interactions with the covariates, the hierarchical structure of the data must be considered. It is important to mention that the data are not independent, but dependent. Thus, multilevel modeling (MLM) instead of regular regression was implemented. MLM has several advantages, for example, it is especially suited for experimental studies with repeated measures, allows incomplete data to still be included in the analysis, and allows examining nested observations (for more information, see Hoffman and Rovine, 2007). In this study, the repeated measurements of the affective states, social interaction intensities, and greenness represented level 1 (situation-level) and were nested within the participants (person-level), representing level 2. Mixed-effects multilevel modeling with restricted maximum likelihood (REML, iterative process) estimations was implemented using R and the corresponding lmer() function from the lme4 package (alpha level *p* < 0.05). Tables for reporting the findings of the final models were generated using the ‘sjPlot’ package (Lüdecke, 2021).

To investigate our hypotheses for each of the three affective states, we conducted separate series of multilevel models in the following step-up approach: First, a null model was conducted, and the variance components were extracted to check for intraclass correlation coefficient (ICC), determining how much of the variation is explained by between- vs. within-person level. Next, the predictors were added consecutively at the situational level, to test the direct effects of social interaction intensities (SI) and greenness (Green) on the affective states. Next, we tested whether random slopes significantly improve the model fits. Finally, the control variables (Sex and Age) and interaction terms (SI*Green) were added consecutively to each of the three affect models to further test our hypotheses. Note: As neither adding random slopes nor adding the interactions improved the model fit, they were both excluded from the equations depicted below. In sum, this led to the following equations (the final equations depict the for all outcome variables best model, a random intercept, fixed slope model):


(1) $$\mathrm{Level}\;1:\;Y_{ti}=b_{0i}+b_{1i}\left(SI\right)+b_{2i}\left(\mathrm{Green}\right)+r_{ti}$$

(2) $$\mathrm{Level}\;2\;\left(\mathrm{Intercept}\right):\;b_{0i}=\gamma_{00}+\gamma_{01}\left(\mathrm{Sex}\right)+\gamma_{02}\left(\mathrm{Age}\right)+\mu_{0i}$$

On level 1 within-participant effects are calculated. (E1) shows the response of a participant (subscript*_i_*) for either of the three subscales (*Y_ti_*) for any e-diary case (subscript _t_). The average intercept of one subscale of affective states for all participants (*b_0i_*) and the predictors from the situation level (level 1) is depicted as Y*_ti_*. The predictors are group-mean-centered with “group” referring to a participant. This enables disaggregation of between- and within-subject effects (Raudenbush and Bryk, 2002). *r_ti_* represents the level 1 (situation level) random error. On Level 2 between-subject effects are assessed, and the fixed-, and random effects and covariates (sex, age; grand-mean-centered) are included. μ_0i_ is the level two random error. Similar to the level 1 random error, the assumption for the random error of level 2 is to be multivariate and normally distributed (expected values of “0” for both). Moreover, all non-significant effects (*p* > 0.05) of the different models were removed to clarify the result presentation.

Furthermore, the level 1 predictors (SI, Green) were standardized, to interpret the degree of their effects on valence, calmness, and energetic arousal, respectively. In addition, as SI was a four-category predictor and to enable interpretation, it was dummy coded: intensity 1 of SI as the reference category, to discriminate and compare situations where participants were alone vs. intensity 2, 3, 4 of SI, which represent situations in which participants experienced social interactions with ascending intensity (Yaremych et al., 2021). This resulted in three dummy variables (intensity 2 = D1, intensity 3 = D2, intensity 4 = D3), depicted in the equations summed as SI for easier reading. The standard deviations were retrieved from the mean of the sample for every predictor from level 1 as well as from the averaged within-participant mean of valence, calmness, and energetic arousal, respectively.

## Results

### Descriptive statistics

The 46 Participants ultimately provided a total of 391 observation data points: Walking-triggered e-diaries revealed 1840 prompts in total, of which 1,206 have been answered, resulting in a compliance rate of 65.5%. However, data condition criteria resulted in a reduction of data usable in the analysis. Note that the majority of data reduction was unavoidable because of the necessity of valid GPS data needed for the determination and calculation of the viewshed green. Also note that the analysis and result presentation is focused on greenness only and not blue spaces. We did not conduct a separate analysis for the blue spaces as only 0.17% (i.e., 0.003 km^2^) of all green and blue spaces in the participants viewsheds in the respective trigger situations were actual blue spaces. We included the few data regarding blue spaces as greenness, because they have similar and comparable associations with mental health (Claßen and Bunz, 2018) (for more information see section ‘Greenness’). Data reduction occurred due to the following: observation points with GPS: 758; GPS accuracy <20 m: 519; within city-boarders: 471; street network range < 30 m: 470; no allocation possible between online questionnaire and AA participants: 391. Ultimately an average of 8,5 e-diary entries per subject per assessment period of 7 days were available (min = 1, max = 48, SD = 10.55). The ICC for valence, calmness, and energetic arousal were ρ_I_ = 0.43, ρ_I_ = 0.48, and ρ_I_ = 0.56, respectively, indicating that 57, 52, and 44% of the variation were caused by within-person level. The variables’ distribution fit the requirements for multilevel analysis. The descriptive statistics for all the variables that have been used in the analysis can be found in Table 1. Note that while not explicitly including walking episodes in the analysis, in 373 of the 391 cases included in the analysis, the following amount of steps were recorded in the 180 s prior to the prompts per participant: mean = 193.27, SD = 95.46. In addition, fixed and random effects of all three subscales of momentary affective states are depicted in Table 1. Next, the main effects of the covariates are described for each subscale of affect separately.

### Affect subscale valence

The following results from the best fit model can be reported (Table 2): Regarding the social interaction intensities during walking episodes, valence was not significantly predicted by the intensities of social interaction participants indicated (D1: *p* = 0.41; D2: *p* = 0.24; D3: *p* = 0.17). This is contrary to our expectations, i.e., no matter the intensity of indicated interaction, participants did not show higher values of valence. Also contrary to our expectations, greenness was not a significant predictor of valence (*p* = 0.71). Furthermore, no interaction effects were found between social interaction intensities and greenness, and none between the fixed effects and the covariates. No differences were found concerning age and none between men and women.

**Table 2:** Multilevel-model analysis results for the associations of social interaction intensity (D1-D3) and greenness (Green) on the affective state dimension valence.

Valence					
Predictors	Estimates	std.Beta	CI	Standardized CI	*p*
(Intercept)	0.52	−0.07	−0.03–1.07	−0.31–0.16	0.064
D1	−0,10	−0.05	−0.35–0.14	−0.18–0.07	0.412
D2	0.15	0.07	−0.10–0.41	−0.05–0.20	0.247
D3	0.16	0.10	−0.07–0.40	−0.04–0.25	0.170
Green	0.00	0.02	−0.00–0.00	−0.07–0.10	0.715
Sex	−0.34	−0.20	−0.71–0.03	−0.41–0.01	0.068
Age	0.01	0.21	−0.00–0.03	−0.00–0.42	0.051
Random Effects					
σ^2^	0.41				
τ_00Participant_	0.27				
ICC	0.40				
N_Participant_	46				
Observations	391				
Marginal *R*^2^/Conditional *R*^2^	0.096/0.454				

Standardized, (std.); Beta, β; CI, 95% confidence intervals; (p), the level of significance; σ^2^, within-person variance; τ_00Participant_, between-person variance; ICC, intraclass correlation coefficient.

### Affect subscale calmness

Calmness (see Table 3), in accordance with our assumptions, was significantly predicted by both social interaction intensities and greenness during walking episodes, with both a higher intensity of interaction and more greenness resulting in participants indicating to be more calm. But, not all three social interaction intensities showed significant associations, i.e., compared with situations in which participants were alone or interacted shortly with someone (D1: *p* = 0.34; D2: *p* = 0.24), they felt calmer when walking together with someone (D3): The effect for D3 shows that participants, who interacted with someone while walking had a 0.16 (*p* = 0.02) points higher score for calmness than those persons who did not interact. Regarding greenness, an increase of 1 SD of greenness led to an increase in calmness of 0.09 (*p* = 0.01), i.e., more calm participants. Similar to subscale valence, no significant interactions were found. Furthermore, no difference between men and women, and none regarding age were identified regarding the level of calmness, and none for the level 1 effects of the predictors.

**Table 3:** Multilevel-model analysis results for the associations of social interaction intensity (D1-D3) and greenness (Green) on the affective state dimension calmness.

Calmness					
Predictors	Estimates	std. Beta	CI	Standardized CI	*p*
(Intercept)	0.20	−0.04	−0.44–0.84	−0.31–0.23	0.540
D1	0.12	0.06	−0.12–0.36	−0.06–0.19	0.340
D2	0.15	0.07	−0.10–0.40	−0.05–0.20	0.247
D3	0.26	0.16	0.03–0.50	0.02–0.31	0.029
Green	0.00	0.09	0.00–0.01	0.02–0.17	0.019
Sex	−0.07	−0.04	−0.50–0.35	−0.28–0.20	0.728
Age	0.01	0.11	−0.01–0.02	−0.13–0.34	0.386
Random effects					
σ^2^	0.40				
τ_00Participant_	0.40				
ICC	0.50				
N_Participant_	46				
Observations	391				
Marginal *R*^2^/Conditional *R*^2^	0.026/0.509				

Standardized, (std.); Beta, β; CI, 95% confidence intervals; (p), the level of significance; σ^2^, within-person variance; τ_00Participant_, between-person variance; ICC, intraclass correlation coefficient.

### Affect subscale energetic arousal

During the examined walking episodes, both the intensities of social interaction and greenness significantly predicted energetic arousal (see Table 4). But, in this case, the comparison showed a significant association between a different intensity level with energetic arousal: Specifically, the effect for D2 shows that participants, who interacted shortly with someone during their walk had a 0.13 (*p* = 0.02) points higher score for energetic arousal than those who did not interact at all or those who interacted with someone while walking (D1: *p* = 0.22; D3: *p* = 0.19). Regarding greenness, the results show that an increase of greenness of 1 SD led to an increase in energetic arousal of 0.08 (*p* = 0.02), indicating more energized participants. In accordance with the other momentary affective state dimension, we found no significant effects regarding the interactions of the predictors. Last, as for Valence and Calmness, no differences were found between women and men.

**Table 4:** Multilevel-model analysis results for the associations of social interaction intensity (D1-D3) and greenness (green) on the affective state dimension energetic arousal.

Energetic arousal					
Predictors	Estimates	std. Beta	CI	Standardized CI	*p*
(Intercept)	0.29	0.01	−0.44–1.01	−0.24–0.26	0.437
D1	0.16	0.07	−0.09–0.41	−0.04–0.17	0.220
D2	0.31	0.13	0.05–0.57	0.02–0.23	0.021
D3	0.16	0.08	−0.08–0.41	−0.04–0.21	0.191
Green	0.00	0.08	0.00–0.01	0.01–0.15	0.022
Sex	−0.20	−0.09	−0.67–0.28	−0.32–0.13	0.418
Age	0.02	0.21	0.00–0.04	0.01–0.43	0.067
Random effects					
σ^2^	0.43				
τ_00Participant_	0.52				
ICC	0.55				
N_Participant_	46				
Observations	391				
Marginal *R*^2^/Conditional *R*^2^	0.082/0.585				

Standardized, (std.); Beta, β; CI, 95% confidence intervals; (p), the level of significance; σ^2^, within-person variance; τ_00Participant_, between-person variance; ICC, intraclass correlation coefficient.

## Discussion

This study aimed to examine associations between social- and physical environmental factors and affective states during walking episodes in urban areas. In addition, we implemented an innovative AA approach, using a combination of GPS- and walking-triggered e-diaries to identify situations of walking episodes. Concerning the hypothesized associations of this study, mixed findings were reported with the following implications:

Contrary to our hypothesis, the affective state of valence was not significantly associated with the intensity of social interaction. This is not in line with findings from Bernstein et al. (2018), who used a study design with 6 semi-random prompts per day over three days upon which participants indicated social interaction and the pleasantness of that interaction, showing more happiness in situations with social interactions. Another study by Monninger et al. (2022) linked social interactions to higher positive affect, but instead of asking the participants “live” in the situations, they indicated the number and quality of social contacts in the last 2 h prior to a prompt. It follows that the three approaches produce different findings, which leads to questions about the eligibility of the different assessment methods and the comparison of findings. Also contrary to our predictions, no association between valence and greenness in the situations in which e-diaries were triggered was found. A possible explanation for this finding could be that the exposure to greenspace was only measured as a percentage of surrounding green right in the moment of the triggered e-diary. This amount of green can often be little compared to a cumulative greenspace exposure that sums up the amount of green along a walking route or a certain time-span, and thus might not be “enough” to have an effect on individuals’ valence. For example, Tost et al. (2019) did find higher valence ratings for participants that were surrounded by a lot of urban vegetation by analyzing viewshed green, greenspace exposure 5 min prior to a valence rating, as well as exposure to green over the course of a week. Furthermore, the results show that regarding valence neither the effect of intensity of social interaction nor that of greenness depend on each other, as their interaction did not show any significant associations with valence.

Most strikingly, and confirming our hypothesis regarding the affective subscale calmness, our results show significant independent associations with both social interaction intensity and greenness during walking episodes. To be precise, participants, who walked together with someone during their walk (= D3), and those who were walking in a greener environment, indicated to be more calm/relaxed. This association between walking together with someone (= D3) and calmness can be explained by a calming/relaxing and supportive effect of being and interacting with someone familiar. Supporting this, e.g., Smyth et al. (2014) found social support to decrease negative mood and to predict less stress severity, and in addition, Schwanen and Wang (2014) also found a positive effect of (close) social contacts (e.g., friends, companions) during activities on wellbeing. The finding that more environmental green was associated with more calm/relaxed individuals’ is in line with the finding of Nutsford et al. (2013), who found that both observable and usable greenspace in urban environments can protect against mood and anxiety disorders. This indicates that people who live in urban environments that are often characterized by negative environmental influences and stimuli like (traffic-) noise, or dull and monotonous surroundings, profit from both social interactions and greenness by helping individuals to remain- or calm down and be more relaxed. As urban populations continue to grow, such calming effects of the social- and physical environment may help to mitigate overstimulation and ultimately aid as protective factors. Also, as for valence, the interaction of greenness and social interaction level did not show significant associations with calmness. This indicates that these effects might not need to co-occur. However, future studies are needed to research these aspects further.

Regarding the third affective state dimension energetic arousal, we also found significant independent associations, with both a higher intensity of social interaction and more greenness resulting in increased energetic arousal. One might conclude that the identified calming effect of both social interactions and greenness as presented for calmness could mitigate higher arousal levels and thus be counterintuitive to these results, but the following has to be considered: First of all, the momentary affective states scales are semantic differentials (compare with section ‘measures’), i.e., being ‘relaxed’ (calmness) does not mean being ‘tired’ (energetic arousal), or being ‘calm’ does not mean, a person is not full of energy. Thus, a person that feels relaxed in a (green) environment can still be tired or awake. Also, on the one hand, contrary to the findings for calmness, only situations, in which subjects were walking outside and had a short interaction with someone (= D2) were significantly associated with energetic arousal. That implies that a more sudden, unexpected interaction has more of an effect on energetic arousal, compared to a calming/relaxing effect when walking with someone familiar (= D3). On the other hand, supporting evidence for the finding of increased energetic arousal after a short walking bout stems from Reichert et al. (2017), who showed that already non-exercise activity increases energetic arousal. Thus, even though Reichert et al. (2017) investigated a time span of 15 min vs. the 100 m and approximately 1–2 min span in our study, we can report similar findings. Next, the finding that more greenness in the participants’ viewshed led to an increase in energetic arousal can be seen as a consequence of individuals feeling awake, and energized when being in a more green environment, compared to more dull experience in environments with no or less environmental green (= urban areas). Supporting evidence comes from Beute and de Kort (2018), who implemented an ecological momentary assessment to assess how nature (and daylight) effect affect and stress of subjects with and without depression and who found positive associations between the exposure to nature (and daylight) and energy levels. In another study, Markevych et al. (2017), attest greenspace restoring capacities, i.e., greenspace helps to reduce stress, restore attention, and elicit positive emotions in general. But, concerning the small effect of greenness, implications have to be considered with caution, as a stronger effect was expected with regard to findings from other studies (e.g., compare McMahan and Estes, 2015). In future steps, a combination of prompts at the beginning of a walking bout and after several minutes may provide further insight into time-dependencies of the effect of social interactions and greenness on energetic arousal levels.

By implementing a new study design with walking-triggered e-diaries, a novel approach to measure social interactions, and additionally enriching the assessed data with subsequently imputed environmental data, we were able to collect data regarding environmental factors and their association with momentary affective states in walking situations. The knowledge about the feasibility of this data assessment method should be used to collect further data in different residential environments. Also, these assessments should include additional environmental factors (e.g., noise, blue spaces), and PA measures to control for the role PA plays in these associations and to increase the informative value of the results of this study and also, to continuously improve data collection methodology. Besides our study design being feasible, several limitations have to be considered. First, only data from 46 participants, and a total of 391 measurements were included in the analysis, which is a comparatively low number for multilevel analysis. But, calculating the viewsheds instead of simple buffers requires a higher accuracy of the GPS signal. In our analysis, we used a GPS accuracy of at least 20 m. As a consequence, data with a lower accuracy was lost. Within built-up areas, GPS accuracy decreases because tall buildings affect the GPS receiver’s contact with the satellites. For example, Schipperijn et al. (2014) report an average accuracy of 11.5 m while walking within urban canyons, with a standard deviation of 14 m. Second, we did not apply multiple testing correction for the *p*-values, which could theoretically lead to the by chance discovery of significant results; however, as our design is experimental, to do no correction is reasonable (Goeman and Solari, 2011). Also, as is often the case, our study sample consisted of 81% individuals with a high education level, making generalizations difficult. Third, future studies should consider combining the present research approach with the additional inclusion of measurements of blue spaces, if such data is present in the respective locations, as blue spaces have been shown to have comparable positive associations with individuals wellbeing and mental health and thus might add important information (Kistemann and Völker, 2014; Grellier et al., 2017; Claßen and Bunz, 2018). Also, future studies should consider to investigate greenness and social interaction in inactive outside-of-home episodes to compare environmental influences on affective states in active vs. non-active situations. Fourth, albeit no severe restrictions like curfews being in place during the time of data collection, data were collected during the Covid-19 pandemic, which led to many changes in individuals’ daily lives and routines, which could hinder comparability. Also, to identify, whether long-term exposure to certain social- and physical environments leads to the development of chronic conditions, it is necessary to combine short-term data collection (i.e., days, weeks) with long-term data collection (i.e., months, years; Chaix, 2020). Furthermore, with the secondary environmental data used in this study, we cannot specify what kind of green exactly led to the observed effect. That is, we cannot determine that the effect of greenness actually stems only from the context of a green environment, i.e., the observed effect might actually also come from other confounding factors like bird noise or a calm environment and may as well depend on weather conditions and not only on the environmental green itself. Thus, future research should try to incorporate measurement methods that can more specifically identify an effect of green itself and should account for the weather conditions as well.

To conclude, we were able to implement a new data assessment method that allows accounting for social- and physical environmental factors and their impact on momentary affective states right in the corresponding situations in which they are present. Usage of the assessment method of this study together with the findings from this study can aid decision-makers regarding the creation and design of more healthy and livable residential areas. Also, our results support the calls from different research fields for stronger incorporation of social- and environmental factors when planning, designing, and evaluating ways to promote walking/active mobility and public health in urban environments.

## Data availability statement

The raw data supporting the conclusions of this article will be made available by the authors, without undue reservation.

## Ethics statement

The studies involving human participants were reviewed and approved by University of Konstanz (IRB18KN010–004, October 29, 2018). The patients/participants provided their written informed consent to participate in this study. The study fully conformed to the Declaration of Helsinki and the ethics guidelines of the German Psychological Society.

## Author contributions

LB: conception of the manuscript, analysis and interpretation of data, writing original draft, data acquisition. JS: GIS-data acquisition and data analysis, revising the manuscript. MK: overall conception and design of the study, revising the manuscript. CN: overall conception and design of the study, revising the manuscript. All authors were involved in critically revising the manuscript, and have given their approval for submitting the manuscript.

## Funding

This study was part of the AMbit project that was funded by the Deutsche Forschungsgemeinschaft, Germany (grant 421868672). Open Access funding was enabled by the Open Access Publishing Fund of the University of Konstanz.

## Conflict of interest

The authors declare that the research was conducted in the absence of any commercial or financial relationships that could be construed as a potential conflict of interest.

## Publisher’s note

All claims expressed in this article are solely those of the authors and do not necessarily represent those of their affiliated organizations, or those of the publisher, the editors and the reviewers. Any product that may be evaluated in this article, or claim that may be made by its manufacturer, is not guaranteed or endorsed by the publisher.

## Footnotes

^1^ https://www.movisens.com/en/products/movisensXS/

^2^ https://www.movisens.com/en/products/

^3^ https://www.movisens.com/en/products/

^4^ https://www.openstreetmap.org/

^5^ https://www.esri.com/de-de/arcgis/products/arcgis-desktop/resources

^6^ https://www.esri.com/

^7^ https://www.mundialis.de/en/deutschland-2020-landbedeckung-auf-basis-von-sentinel-2-daten/

^8^ https://www.bmvi.de/SharedDocs/DE/Artikel/DG/mfund-projekte/incora.html
